# The application of AI-driven digital phenotyping and interventions in neuropsychiatric disorders

**DOI:** 10.3389/fpsyt.2026.1907262

**Published:** 2026-09-21

**Authors:** Yuhan Liu, D. Logan Lu, Yating Hong, Lianting Hu, Hui Tang, Yaping Cheng, Yongshang Yu, Xuntao Yin, Huimin Xia, Long Lu

**Affiliations:** 1 College of Life Sciences, Wuhan University, Wuhan, China; 2 School of Information Management, Wuhan University, Wuhan, China; 3 Data Center, Wuhan Children’s Hospital, Wuhan, China; 4 Department of Clinical Data Center, Guangzhou Women and Children’s Medical Center, Guangzhou Medical University, Guangzhou, China; 5 Department of Pediatric Rehabilitation, Guizhou Provincial Rehabilitation Hospital, Guiyang, China; 6 Healthcare Big Data Research Center, The Big Data Institute, Wuhan University, Wuhan, China

**Keywords:** artificial intelligence, closed-loop systems, digital mental health, digital phenotyping, neuropsychiatric disorders, personalized intervention

## Abstract

Artificial intelligence (AI) is increasingly influencing the management of neuropsychiatric disorders, extending its role beyond assistive diagnosis toward adaptive intervention and individualized disease management. Digital phenotyping enables continuous assessment of multimodal behavioral and biological signals, allowing neuropsychiatric disorders to be modeled as dynamic rather than static processes. This review summarizes current AI-driven approaches for neuropsychiatric assessment and intervention, including computer vision–based behavioral analysis, wearable sensing, neuroimaging biomarkers, digital therapeutics, virtual reality, social robotics, and brain–computer interfaces. We further discuss emerging closed-loop frameworks that integrate real-time state estimation with adaptive intervention strategies, shifting neuropsychiatric AI from passive observation toward continuous therapeutic optimization. Despite rapid progress, integration between phenotyping and intervention remains limited, and most closed-loop systems are still confined to experimental settings. Key challenges include multimodal representation, interpretability, generalizability across populations and devices, and translation into real-world clinical workflows. We argue that the primary bottleneck in AI-driven neuropsychiatry is no longer prediction accuracy alone, but the ability to connect continuous patient-state estimation with clinically meaningful adaptive action. Finally, we outline future directions toward clinically grounded, human-AI collaborative neuropsychiatric systems.

## 1 Introduction

Neuropsychiatric disorders are among the leading causes of disability and disease burden worldwide (1, 2), including neurodegenerative diseases such as Alzheimer’s disease (AD) and Parkinson’s disease (PD), psychiatric conditions such as major depressive disorder, and neurodevelopmental disorders such as autism spectrum disorder. Although clinically distinct, these conditions share one important feature: variability is not an exception but a defining property. This variability appears at multiple levels. At the clinical level, symptom expression differs widely even among patients carrying the same diagnosis. At the biological level, differences in neural circuitry, neurotransmitter dynamics, and compensatory mechanisms contribute to divergent disease trajectories. These layers of heterogeneity do not align neatly with current diagnostic categories.

Longitudinal cohort studies have repeatedly shown that disease progression is better captured through temporal evolution than through baseline severity scores alone. In some cases, early dynamic changes outperform static measurements in predicting long-term outcomes (3, 4). Despite this, clinical practice still relies heavily on episodic assessment. Patients are typically evaluated at discrete time points separated by weeks or months, creating a mismatch between continuous disease dynamics and sparse observation (5). AI-based digital phenotyping attempts to bridge this gap by integrating multimodal signals into unified patient state representations. Examples include behavioral, physiological, and neuroimaging data streams collected in naturalistic settings (6).

Recent advances in sequential modeling and reinforcement learning have provided conceptual frameworks for moving neuropsychiatric intervention beyond static protocols toward adaptive decision-making (7, 8). However, neuropsychiatric disorders are only partially observable in practice, and clinical outcomes are often delayed, heterogeneous, and context-dependent. Whether computational optimization can be meaningfully aligned with clinical reasoning therefore remains unresolved (8). Recent studies have attempted to address this challenge by improving the characterization of neuropsychiatric states through continuous analysis of complex behavioral and biological signals (6). Accept conventional machine learning models, emerging AI approaches, including transformer-based architectures and advanced representation learning methods, have been explored for more effective extraction of clinically relevant patterns from high-dimensional biomedical data (9–11). These approaches have been applied to behavioral monitoring, physiological sensing, and neuroimaging analysis, providing new opportunities for identifying clinically meaningful patterns and constructing more individualized representations of disease states. This review argues that the major challenge in AI-driven neuropsychiatry is not prediction accuracy per se, but the integration of continuous state estimation with clinically meaningful adaptive intervention.

## 2 AI-Driven digital phenotyping assessment

Digital phenotyping aims to extend neuropsychiatric assessment into real-world environments by integrating behavioral, physiological, and neuroimaging data streams (12). While this enables richer disease state representations, it also introduces structural and technical challenges (6, 13). Data collection parameters—sampling rate, sensor configuration, missing data—directly constrain analytical models and research questions. Therefore, acquisition and analysis must be integrated (5, 6).

### 2.1 Behavioral phenotyping based on computer vision

Clinician observation and rating scales remain the historical backbone of behavioral assessment, but they suffer from subjectivity and poor temporal resolution (14, 15).

Computer vision enables automated quantification of observable behaviors from video data. By combining facial expression analysis, pose estimation, gaze tracking, and action recognition with deep learning algorithms, it transforms complex behavioral signals into objective and continuous digital biomarkers. Compared with clinician-rated behavioral assessment, computer vision provides higher temporal resolution, improved reproducibility, and scalability for large-scale monitoring.Recent deep learning models, including convolutional neural networks and transformer-based architectures, can capture subtle facial and motor dynamics such as micro-expression timing, gaze shifts, and fine-grained gait irregularities (16).

Multicenter studies have further demonstrated promising performance in autism spectrum disorder (ASD) classification or screening tasks (17, 18). Computer vision approaches have also been widely explored for movement disorders such as PD. Frameworks like OpenPose have been adopted to quantify motor signs. In a recent study, the correlations between pose-derived gait metrics and Unified PD Rating Scale (UPDRS) motor subscores have shown moderate associations with clinical motor severity measures, depending on the specific motor task and patient cohort. Correspondingly, the classification performance for differentiating PD from healthy controls in those studies can reach around 80%–90% under controlled laboratory protocols (19, 20).

The limitation is that performance in real-world environments tends to be noticeably lower. Studies using smartphone-based video capture have reported relative drops when moving from controlled to naturalistic settings. One study also reported that a model achieving 91% training accuracy on controlled video dropped to 69% when tested on smartphone-captured real-world data—a decrease of 22% (21).A more fundamental limitation is context dependence, which means identical motor or facial patterns may reflect different underlying conditions. For example, resting tremor in PD (basal ganglia dysfunction) versus action-induced tremor in essential tremor (cerebello-thalamo-cortical circuitry) (22, 23).

### 2.2 Physiological signals and wearable data

Physiological signals and wearable sensing technologies provide continuous access to dynamic changes in autonomic, motor, and neural states, representing an important component of AI-driven digital phenotyping. Commonly collected signals include heart rate variability (HRV), electrodermal activity (EDA), electroencephalography (EEG), and motion-related parameters obtained from accelerometers or inertial measurement units (24). Compared with clinician-based assessments conducted at discrete time points, wearable sensing enables longitudinal monitoring in naturalistic environments and captures temporal fluctuations that may be overlooked during routine clinical visits. By integrating these signals with machine learning models, physiological data can be transformed into digital biomarkers reflecting disease progression, symptom variability, and treatment response (25).

Wearable-based physiological monitoring has been increasingly investigated across several neuropsychiatric and neurological disorders. In PD, accelerometer and inertial sensor data have been used to quantify motor symptoms, including tremor, gait disturbance, and daily activity fluctuations, providing objective measurements beyond conventional clinical rating scales (26). In epilepsy, EEG-based wearable systems aim to detect abnormal neural activity and support seizure prediction or early warning (27). Beyond disease monitoring, continuous physiological sensing has also shown potential for evaluating treatment effects by tracking changes before and after therapeutic interventions.

However, in the actual application scenarios of this technology, heterogeneity in hardware, sampling protocols, and preprocessing pipelines introduces often-underestimated variability (28). As Onnela notes (5), missing real-world data are rarely random but often informative about user behavior or device state; ignoring this can bias state estimation and impair generalization.

### 2.3 Neuroimaging and AI-derived biomarkers

Neuroimaging remains one of the most informative modalities for studying neuropsychiatric disorders, enabling characterization of brain organization at multiple biological levels, including anatomy, functional connectivity, and network dynamics. Deep learning models have shown strong performance in detecting disease-related structural and functional abnormalities, particularly in AD (9, 10).In AD classification tasks based on Alzheimer’s Disease Neuroimaging Initiative(ADNI) datasets, reported accuracies are frequently high (often >85%), depending on preprocessing pipelines and feature selection strategies (29).

Beyond diagnosis, neuroimaging-based AI models have also been explored for disease progression monitoring and prognosis prediction. Longitudinal studies have shown that alterations in brain structure and functional connectivity may provide predictive information regarding cognitive decline and disease trajectory. In addition, network-based analyses, including graph neural network approaches, enable modeling of complex interactions among distributed brain regions, providing new opportunities for identifying system-level biomarkers instead of isolated regional abnormalities (30).

Despite substantial progress, several challenges limit the clinical implementation of AI-derived neuroimaging biomarkers. First, model generalizability remains a major obstacle. Neuroimaging datasets often vary across institutions due to differences in scanner platforms, acquisition parameters, preprocessing pipelines, and demographic characteristics, leading to performance degradation when models are transferred from research cohorts to external clinical populations (31). Furthermore, many neuroimaging studies are based on relatively small and homogeneous datasets, which may limit the assessment of reproducibility, generalizability, and potential clinical utility (32).

## 3 AI-assisted intervention strategies

Compared with assessment systems, AI-driven intervention frameworks are still at an earlier stage of development. Most existing approaches demonstrate adaptive behavior in controlled environments, but clinical validation remains limited. High prediction accuracy in disease detection or symptom monitoring also does not automatically translate into effective intervention. A system that reliably estimates tremor severity may still fail to guide adaptive stimulation that improves real-world motor function. Thus, the critical gap is not better predictors, but closed-loop systems that link estimation to clinically meaningful action (33).

As summarized in **Figure 1**, current AI applications in neurology span multiple disorders and intervention modalities, with substantial differences in technological maturity and clinical integration across disease domains.

**Figure 1:**
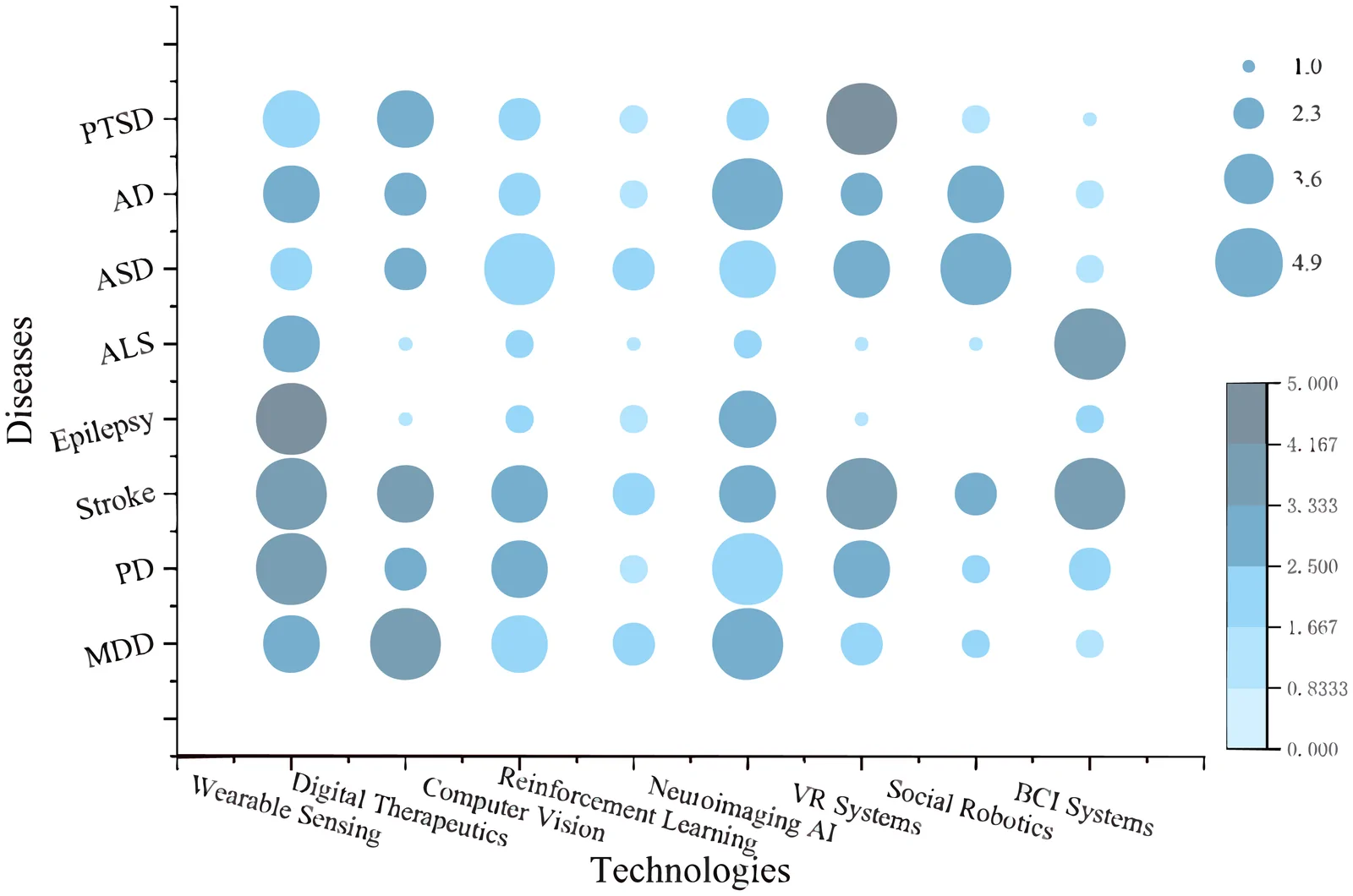
AI applications across neurological disorders and adaptive intervention modalities. Different neurological disorders exhibit heterogeneous patterns of AI technology integration across diagnostic assessment, digital phenotyping, biomarker discovery, and adaptive intervention frameworks. The bubble matrix summarizes the evidence landscape of AI-assisted technologies across major neurological and neuropsychiatric disorders. Each bubble represents the association between a specific AI modality and disorder, with bubble size indicating the strength of available evidence, based on the extent of published research, methodological validation, and accumulated evidence supporting the application of the corresponding technology. Color intensity represents translational maturity, reflecting the degree of clinical validation and practical implementation, ranging from early exploratory research to more established clinical applications. Neurodegenerative disorders, including Parkinson’s disease (PD) and Alzheimer’s disease (AD), demonstrate strong associations with wearable sensing, neuroimaging-based artificial intelligence, computer vision–enabled behavioral assessment, and adaptive rehabilitation technologies. Psychiatric and neurodevelopmental disorders, such as major depressive disorder (MDD), post-traumatic stress disorder (PTSD), and autism spectrum disorder (ASD), show increasing integration with digital therapeutics, virtual reality (VR), computer vision, and social robotics for personalized behavioral assessment and intervention. Neurological conditions including epilepsy, stroke, and amyotrophic lateral sclerosis (ALS) exhibit prominent associations with wearable monitoring, brain–computer interface (BCI) systems, and adaptive control frameworks. The figure illustrates the uneven yet interconnected landscape of AI applications, highlighting that research intensity and clinical translation maturity do not always progress in parallel across different disease–technology combinations.

### 3.1 Digital therapeutics

Digital therapeutics deliver structured, evidence-based interventions via software platforms, often adjusting task parameters based on user performance metrics such as accuracy or response time. Compared with some conventional interventions, digital therapeutics may provide low marginal cost, high individualization, and the ability to overcome temporal, spatial, and human resource constraints—advantages that have contributed to increasing interest and clinical exploration in neuropsychiatric disorders (34).

Digital therapeutics have been increasingly applied across a range of neuropsychiatric disorders, particularly conditions characterized by persistent symptoms, behavioral dysfunction, or the need for long-term self-management. In depressive disorders, digital cognitive behavioral therapy platforms provide structured psychological interventions by delivering cognitive restructuring, behavioral activation, and symptom monitoring through software-based systems (35). In mild cognitive impairment and early-stage dementia, computerized cognitive training programs are also applied to maintain or improve cognitive function through repetitive and adaptive training tasks (36).

Clinical evidence, however, remains mixed. In mild cognitive impairment, cognitive training produces only modest short-term gains in executive function, with inconsistent evidence for long-term functional benefit (37). For depression, digital cognitive behavioral therapy can achieve short-term symptom reduction comparable to conventional therapy, yet dropout rates exceeding 40% have been reported in some specific studies, underscoring a persistent adherence challenge (38).

### 3.2 AI-enhanced VR interventions

Virtual reality (VR) provides controlled and reproducible environments for cognitive and behavioral training. Recent AI-assisted VR systems further extend this framework by incorporating real-time behavioral and physiological state estimation into adaptive intervention loops. Machine learning models can integrate multimodal signals such as heart rate variability, electrodermal activity, gaze behavior, and movement patterns to estimate anxiety level, emotional arousal, or cognitive load during virtual interaction. Based on these estimated states, the system can dynamically adjust exposure intensity, task difficulty, or environmental complexity in real time, whose strength lies in the ability to systematically manipulate environmental conditions while maintaining experimental control (39). Yet the controllability of virtual environments also creates an important trade-off: experimental consistency is gained partly by reducing the unpredictability and sensory complexity of real-world interaction (40, 41). In post-traumatic stress disorder (PTSD) interventions, VR-based exposure therapy has shown beneficial effects on avoidance behavior and emotional regulation in selected populations. In this sense, AI functions not merely as an analytical tool, but as the adaptive control layer that links continuous patient-state estimation to personalized intervention adjustment. Yet outcomes remain heterogeneous across studies (39, 42).

### 3.3 Social robotics and communication systems

Social robots and AI-based communication systems are designed to support structured interaction training in populations with impaired communication abilities (43). In autism spectrum disorder interventions, robot-assisted training has been associated with increased engagement and task participation compared with traditional human-mediated sessions in some studies (44, 45). Yet current evidence suggests that the structural simplicity of robotic interactions may limit transfer to natural social environments, and long-term generalization remains insufficiently characterized (44, 46).

### 3.4 AI-driven brain–computer interfaces

Brain–computer interfaces (BCIs) establish a direct link between neural activity and external systems by decoding brain signals. Current BCIs mainly include non-invasive approaches based on EEG and invasive systems using implanted electrodes. The integration of AI algorithms has improved neural signal decoding and enabled more adaptive systems that can better capture individual brain states. For example, machine learning and deep learning models are widely used to classify and interpret EEG or intracortical neural activity patterns, transforming noisy high-dimensional signals into behaviorally meaningful outputs (47, 48).

Most clinical applications of BCIs have focused on neurological rehabilitation and communication assistance. In stroke and motor disorders, BCI-based rehabilitation has shown potential in promoting motor recovery through neurofeedback training (49). For patients with severe motor impairment, such as ALS, BCIs provide alternative communication pathways (50).

Reported classification accuracies are high in controlled settings, although performance varies substantially across individuals. Future research will likely combine AI-based decoding with multimodal sensing technologies to develop more stable and personalized closed-loop intervention systems (51, 52).

Overall, current AI-assisted intervention technologies differ substantially in their data modalities, adaptive mechanisms, and clinical maturity. Importantly, the role of AI varies across systems, ranging from behavioral feature extraction and physiological state estimation to neural decoding and sequential intervention optimization(**Table 1**).

**Table 1:** Comparison of AI-assisted neurological assessment and intervention systems.

Technology	Disease	Clinical use	Human evidence	Major limitation	Future potential
Computer vision	PD	Motor assessment	Human observational studies	Context dependence	Longitudinal monitoring
Computer vision	ASD	Behavioral assessment/screening	Human studies, including multicenter studies	Generalizability	Objective behavioral assessment
Wearable sensing	PD	Motor fluctuation monitoring	Human longitudinal studies	Device heterogeneity	Continuous monitoring
Wearable sensing	Epilepsy	Seizure detection/ prediction	Human Studies	False positives	Closed-loop warning/stimulation
Neuroimaging AI	AD	Diagnosis/prognosis	Large research cohorts	Domain Shift	Clinical decision support
Digital therapeutics	Depression	Symptom intervention	RCT evidence	Adherence	Personalized intervention
VR	PTSD	Exposure therapy	Clinical Studies	Ecological validity	Personalized exposure
Social robotics	ASD	Social communication training	Human pilot/clinical studies	Limited generalization	Adaptive social intervention
BCI	Stroke	Motor rehabilitation	Human clinical studies	Inter-subject variability	Closed-loop rehabilitation
BCI	ALS	Communication	Human Studies	Signal stability	Assistive communication
Reinforcement learning	Multiple	Adaptive intervention	Mostly experimental	Reward/causality	Closed-loop treatment

## 4 Integration of assessment and intervention

In contemporary clinical neuropsychiatry, assessment and intervention are still largely organized as two temporally and conceptually separated stages. Data are first collected through clinical visits, laboratory tests, or digital monitoring systems, and then interpreted retrospectively to guide treatment decisions (53). Although this workflow is well established in conventional medical practice, it is fundamentally misaligned with the dynamic and continuously evolving nature of neuropsychiatric disorders.

Conditions such as PD, epilepsy, and major depressive disorder often exhibit non-stationary symptom trajectories, where patient states fluctuate over hours, days, or longer disease phases (54–56). As a result, discrete assessment points provide only partial and sometimes misleading representations of underlying disease dynamics. This mismatch has motivated closed-loop neuropsychiatric systems that integrate assessment, interpretation, and intervention into a continuous feedback cycle. Unlike traditional linear pipelines, closed-loop systems treat patient state estimation and treatment adjustment as coupled processes rather than sequential steps (57).

As illustrated in **Figure 2**, AI-driven closed-loop neurological systems integrate multimodal sensing, continuous state estimation, adaptive intervention generation, and feedback updating into a unified dynamic framework.

**Figure 2:**
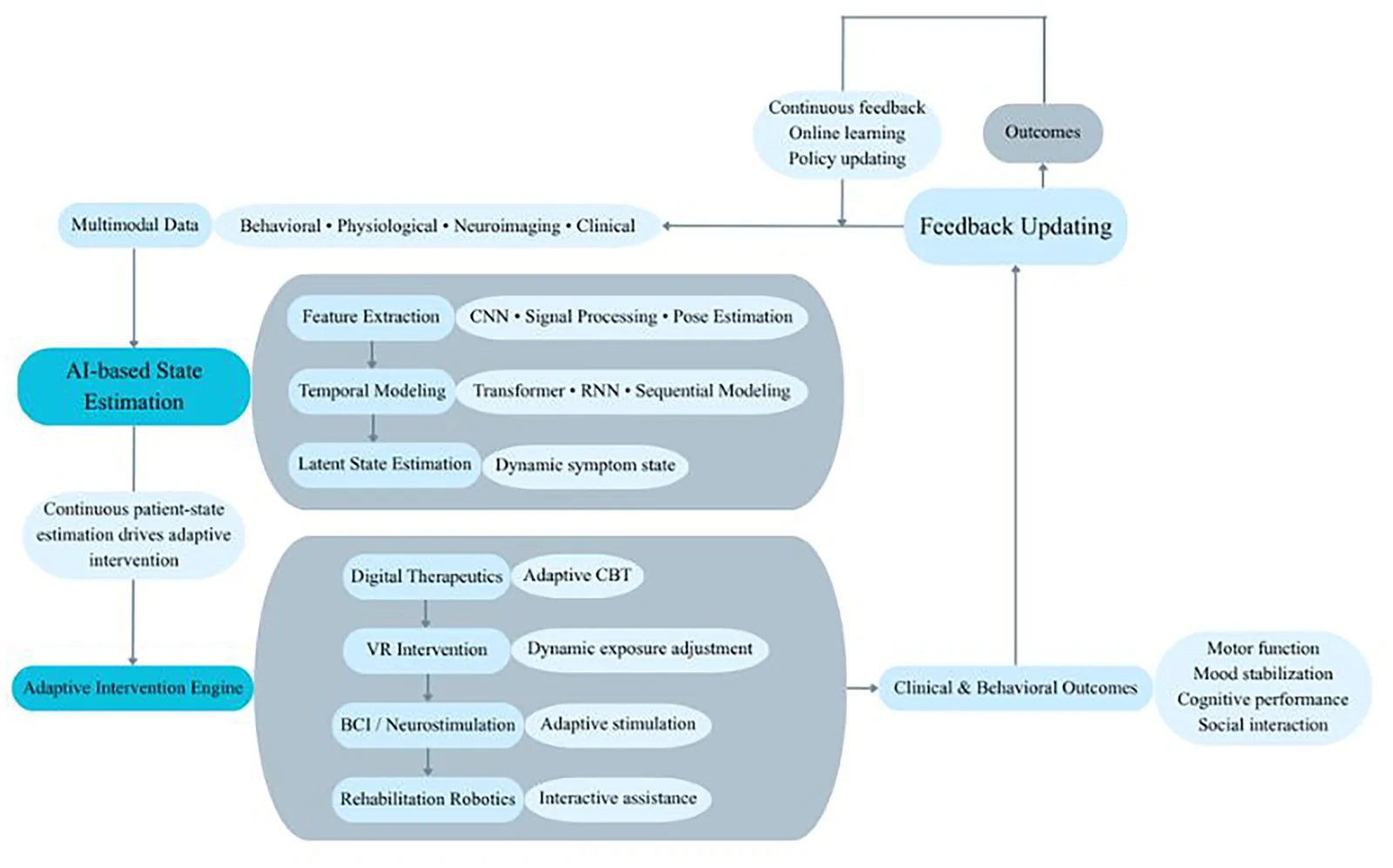
Conceptual architecture of AI-driven closed-loop neurological systems. Multimodal behavioral, physiological, neuroimaging, and clinical data are continuously integrated for real-time patient-state estimation. AI models generate adaptive intervention strategies based on estimated disease states and continuously update intervention policies through feedback-driven learning mechanisms.

### 4.1 Conceptual architecture of closed-loop neuropsychiatric systems

From a systems perspective, AI-driven closed-loop frameworks in neuropsychiatry typically consist of four interdependent modules: (1) multimodal data acquisition, (2) real-time state estimation, (3) intervention policy generation, and (4) feedback-based model updating (53, 57, 58).

Data acquisition integrates heterogeneous signals, including behavioral data (speech, movement, facial expression), physiological signals (EEG, HRV, EDA), and in some cases neuroimaging-derived biomarkers. These data streams differ significantly in temporal resolution and noise characteristics, requiring modality-specific preprocessing pipelines before integration (12, 59).

State estimation is typically performed using machine learning models that construct latent representations of disease status. In recent studies, transformer-based architectures and temporal convolutional networks have been increasingly used to capture long-range dependencies in physiological and behavioral sequences (60). In PD monitoring systems, for example, such models have been used to estimate motor state fluctuations in near real-time based on wearable sensor input. The intervention module maps estimated states to treatment actions. These actions may include cognitive task adaptation, medication suggestions, stimulation protocols, or behavioral interventions depending on the clinical context. Finally, feedback updating enables continuous refinement of both state estimation and intervention policies based on observed outcomes (57, 61). This feedback loop lies at the heart of the challenge: without it, high prediction accuracy remains clinically underutilized.

### 4.2 Reinforcement learning as a decision-making framework

Reinforcement learning (RL) has emerged as one of the most commonly proposed frameworks for implementing closed-loop neuropsychiatric systems, due to its natural formulation of sequential decision-making under uncertainty (8, 62). In neuropsychiatric applications, reinforcement learning reframes intervention as a sequential optimization problem in which treatment decisions are continuously adjusted according to estimated patient state and observed outcomes. Over time, the system learns a policy that aims to maximize cumulative long-term reward rather than short-term performance improvement. This formulation is particularly relevant for neuropsychiatric disorders, where therapeutic effects are often delayed and nonlinear (8). In cognitive training for mild cognitive impairment, immediate task performance may improve without corresponding long-term functional gains, indicating a mismatch between short-term optimization signals and clinically meaningful outcomes (63).

A central unresolved issue is partial observability and uncertain causality. Clinical outcomes depend on medication adherence, comorbidities, and environmental context — not just the intervention (8, 64). Optimizing purely on observed correlations may therefore not capture clinically meaningful improvement. Counterfactual reasoning and off-policy evaluation offer potential ways to estimate intervention effects from incomplete trajectories, but reliable causal modeling in real-world neuropsychiatry remains highly challenging (65).

### 4.3 Emerging experimental evidence of closed-loop systems

Early experimental studies have demonstrated the feasibility of partial closed-loop systems in specific neuropsychiatric domains.

In PD, wearable-based adaptive stimulation systems have been explored, where motor symptom severity estimated from accelerometer data is used to dynamically adjust rehabilitation intensity. Preliminary studies suggest that such adaptive systems have been proposed and investigated compared with fixed-intensity protocols, although evidence remains limited to small cohorts (58, 66).

In depression research, mobile-based ecological momentary assessment (EMA) systems combined with adaptive cognitive behavioral interventions have shown potential in tracking mood fluctuations and adjusting intervention timing. Some longitudinal studies report improved symptom stabilization compared with non-adaptive digital interventions, although effect sizes vary significantly across populations (34).

In epilepsy, EEG-based seizure prediction systems have been integrated with warning or stimulation mechanisms in experimental settings. These systems aim to provide pre-emptive intervention signals minutes before seizure onset. However, real-world deployment remains constrained by false positive rates and inter-patient variability (67, 68).

Overall, closed-loop systems are technically feasible, but most still fall short of integrating continuous state estimation with adaptive, clinically meaningful intervention—the central challenge we identify in this review.

## 5 Challenges

Despite rapid progress in AI-driven digital phenotyping and intervention systems, translation into routine neuropsychiatric practice remains constrained by a combination of methodological, clinical, and infrastructural limitations. These challenges—multimodal heterogeneity, interpretability, domain shift, and governance—each contribute to the same bottleneck: preventing prediction accuracy from becoming clinically actionable intervention.

### 5.1 Multimodal heterogeneity and representation instability

A central difficulty lies in the intrinsic heterogeneity of multimodal neuropsychiatric data. Behavioral, physiological, and neuroimaging signals differ not only in sampling frequency and dimensionality but also in their underlying generative mechanisms.

For example, wearable-derived heart rate variability reflects autonomic regulation at a second-to-minute scale, whereas neuroimaging captures structural or functional organization over much longer biological timescales (22, 24).These mismatched temporal resolutions complicate joint representation learning, particularly in models that assume synchronized inputs (69). In practice, multimodal fusion models often rely on late fusion or latent alignment strategies, which implicitly assume comparability between modalities (11). However, empirical studies in multimodal depression and PD datasets have shown that naive fusion can lead to unstable performance gains, and in some cases even degrade predictive accuracy when one modality is noisy or missing (5). This suggests that the challenge is not simply integration, but representation compatibility across fundamentally different biological signals.

### 5.2 Interpretability gap in clinically meaningful decision-making

Deep learning models achieve high predictive accuracy, but their internal representations often lack direct clinical meaning (70, 71).

In neuroimaging, for instance, convolutional models may identify distributed voxel-level patterns associated with AD classification, yet these patterns do not always map cleanly onto known pathological mechanisms such as tau accumulation or amyloid spread. Similarly, in wearable-based depression monitoring, latent embeddings may correlate strongly with symptom severity but provide limited insight into which physiological processes drive the prediction (72, 73). For neuropsychiatric disorders, predictive performance alone is rarely sufficient. Clinical adoption depends on whether model behavior can be related to recognizable disease mechanisms, progression patterns, or treatment rationale (74).

### 5.3 Generalization failure under domain shift

Generalization across sites, populations, and devices remains a major unresolved issue. Across neuroimaging studies, performance drops are commonly observed when models trained on single-site datasets are evaluated on external cohorts (75–77). This degradation is not limited to imaging. Wearable sensor models also exhibit significant performance instability when transferred across hardware platforms or demographic groups. Even minor differences in sampling frequency or sensor placement can lead to measurable shifts in feature distributions (28).

A more subtle issue is population stratification. Models trained on relatively homogeneous cohorts may not generalize to different age structures or cultural contexts, which is particularly relevant in psychiatric and neurodevelopmental disorders (78, 79).

### 5.4 Ethical, privacy, and governance constraints

Unlike traditional clinical data, digital phenotypes may capture fine-grained behavioral signatures of individuals’ daily lives, including mobility patterns, social interaction frequency, and sleep behavior (80). This raises concerns not only about data privacy but also about secondary use and inference risks: even anonymized datasets may allow re-identification when combined with multimodal contextual information (81).

In addition, algorithmic bias remains an underexplored issue in neuropsychiatric AI systems. Performance disparities across gender, age, and socioeconomic groups have been reported in several studies, particularly in speech- and behavior-based models. Importantly, these biases are often not intentional but emerge from dataset composition and collection environments. Addressing them requires systematic auditing rather than *post hoc* correction (79, 82).

## 6 Future directions

Further improvements in individual model architectures alone are unlikely to resolve the major barriers limiting clinical translation. The future of AI-driven neuropsychiatric systems will likely depend on advances in data infrastructure, learning strategies, and pathways for clinical integration. Several emerging applications illustrate early progress toward these goals. In ASD assessment, AI-based behavioral analysis has demonstrated feasibility in extracting behavior-related features relevant to clinical evaluation (17), while digital phenotyping platforms have enabled large-scale monitoring of mood and behavioral patterns (6). These emerging studies highlight a gradual transition from isolated predictive models toward clinically oriented, multimodal, and interpretable AI systems. Nevertheless, achieving routine clinical implementation will require further advances in data harmonization, external validation, and human–AI integration (33).

### 6.1 Longitudinal multimodal dataset construction

Large-scale longitudinal datasets can capture disease evolution across multiple modalities and time scales. Existing datasets such as ADNI and Parkinson’s Progression Markers Initiative(PPMI) have demonstrated the value of longitudinal imaging and clinical follow-up, but integration with real-world wearable and behavioral data remains limited (3, 83). Future datasets should not only expand sample size but also improve temporal density, enabling modeling of intra-day and inter-day variability in neuropsychiatric symptoms (4, 5). More importantly, data standardization across centers remains as critical as data collection itself. Without harmonized protocols for preprocessing and annotation, increasing dataset size alone does not resolve generalization issues (13, 75, 84).

### 6.2 From predictive models to adaptive systems

Most existing neuropsychiatric AI systems still function primarily as observational models: they estimate disease state but do not meaningfully participate in therapeutic adaptation (33, 71). Recent predictive models have demonstrated increasing ability to forecast disease conversion trajectories, such as progression from mild cognitive impairment to Alzheimer’s disease, although translation into adaptive intervention remains limited (85). A major future shift will involve transitioning from static prediction models to adaptive systems that continuously update based on patient response. This requires tighter coupling between inference and intervention modules (86).

Reinforcement learning and online learning frameworks provide theoretical foundations for this transition (8, 64). However, their clinical application remains limited by reward design ambiguity. Unlike simulation environments, clinical outcomes are delayed, multi-dimensional, and often partially observable. This makes direct optimization non-trivial and explains why most current systems remain constrained to semi-adaptive behavior (8, 65).

### 6.3 Human–AI co-adaptive clinical ecosystems

Compared with replacing clinicians, future AI systems are more likely to function as co-adaptive partners within clinical workflows (7). This requires a shift in system design from standalone algorithms to integrated decision-support ecosystems. In such systems, clinicians provide contextual judgment, while AI systems offer continuous monitoring, pattern detection, and early warning signals. The interaction is bidirectional: clinician feedback also updates model behavior over time (86).

### 6.4 Toward clinically grounded closed-loop neuropsychiatry

Unlike purely computational closed-loop models, clinically viable systems must operate under constraints of interpretability, safety, and accountability. Beyond predictive performance, uncertainty estimation is increasingly recognized as a critical component for safe clinical deployment of AI systems, particularly in high-stakes decision environments where model confidence may directly influence intervention strategies (87). This implies that future systems will need to explicitly encode clinical priors, quantify and communicate predictive uncertainty (70, 76), and allow clinician override at multiple decision layers. Rather than aiming for fully autonomous systems, the more realistic trajectory is toward semi-autonomous, continuously updating clinical decision infrastructures that support human–AI collaborative decision making (71, 88).

### 6.5 Foundation models and multimodal clinical agents

Foundation models learn shared representations across heterogeneous modalities — an appealing approach for neuropsychiatry, where clinically relevant information is often fragmented across imaging, physiology, and clinical records (7, 10, 71). This may be particularly relevant for neuropsychiatric disorders, where clinically meaningful information is often fragmented across imaging, physiological signals, behavioral patterns, and longitudinal clinical records.

Large language models can process unstructured clinical text, including physician notes and patient-reported symptoms (7, 89). Emerging multimodal systems further combine imaging, wearable sensing, and bio-signal data within unified analytical frameworks. In disorders such as PD or depression, these models may help capture symptom fluctuations that are difficult to observe during routine clinical visits.

Interest has also grown in agent-based systems that operate over longer clinical time scales rather than generating isolated predictions. Such systems may eventually assist with progression monitoring and adaptive intervention decisions. At the same time, important limitations remain. AI systems trained on non-representative datasets may reproduce or amplify existing biases (78, 79), and their internal representations are often difficult to interpret in clinically meaningful terms (70, 88). For heterogeneous neuropsychiatric disorders, practical clinical value will likely depend more on reliability and clinical integration than on model scale itself.

## 7 Conclusion

AI-driven digital phenotyping provides a foundation for understanding neuropsychiatric disorders as continuously evolving processes rather than isolated clinical states. Intervention systems are evolving along the same trajectory, although their clinical maturity remains lower. Closed-loop frameworks represent a potential transition from passive monitoring toward adaptive disease management, but their clinical implementation requires stronger evidence for reliability, generalizability, and safety. The long-term significance of AI in neuropsychiatry may not lie in replacing clinical expertise, but in transforming care from episodic observation toward personalized and clinically integrated management. Future systems will likely depend on their ability to bridge this gap by connecting patient-state estimation with interventions that are adaptive, clinically meaningful, and aligned with medical reasoning.
